# Why Do Citizens Share COVID-19 Fact-Checks Posted by Chinese Government Social Media Accounts? The Elaboration Likelihood Model

**DOI:** 10.3390/ijerph181910058

**Published:** 2021-09-24

**Authors:** Qiang Chen, Yangyi Zhang, Richard Evans, Chen Min

**Affiliations:** 1 School of Journalism and New Media, Xi’an Jiaotong University, Xi’an 710049, China; hustcq@163.com (Q.C.); zyy1998@stu.xjtu.edu.cn (Y.Z.); 2 Faculty of Computer Science, Dalhousie University, Halifax, NS B3H 4R2, Canada; richard.evans@brunel.ac.uk; 3 College of Public Administration, Huazhong University of Science and Technology, Wuhan 430074, China; 4 Department of Media and Communication, City University of Hong Kong, Hong Kong 999077, Hong Kong

**Keywords:** COVID-19 misinformation, government social media, fact-checking, elaboration likelihood model, information diffusion

## Abstract

Widespread misinformation about COVID-19 poses a significant threat to citizens long-term health and the combating of the disease. To fight the spread of misinformation, Chinese governments have used official social media accounts to participate in fact-checking activities. This study aims to investigate why citizens share fact-checks about COVID-19 and how to promote this activity. Based on the elaboration likelihood model, we explore the effects of peripheral cues (social media capital, social media strategy, media richness, and source credibility) and central cues (content theme and content importance) on the number of shares of fact-checks posted by official Chinese Government social media accounts. In total, 820 COVID-19 fact-checks from 413 Chinese Government Sina Weibo accounts were obtained and evaluated. Results show that both peripheral and central cues play important roles in the sharing of fact-checks. For peripheral cues, social media capital and media richness significantly promote the number of shares. Compared with the push strategy, both the pull strategy and networking strategy facilitate greater fact-check sharing. Fact-checks posted by Central Government social media accounts receive more shares than local government accounts. For central cues, content importance positively predicts the number of shares. In comparison to fact-checks about the latest COVID-19 news, government actions received fewer shares, while social conditions received more shares.

## 1. Introduction

In the information age with relaxed freedom of speech, the spread of misinformation through social media platforms poses one of society’s greatest challenges. During health emergencies, such as the COVID-19 outbreak, citizens flock to social media to seek health-related information. Channels, such as Facebook and Sina Weibo, provide citizens with an overabundance of information, some of which factual (i.e., verified and evidence-based) and some not. As the pandemic evolves, killing over 2.75 million people worldwide, as of 26 March 2021, the World Health Organization (WHO) warned that society is faced with a correlated pandemic, termed ‘infodemic’, which describes the prevalence of misinformation, rumors, and uncertainty, being spread on social media [1,2]. Misinformation (i.e., claims that have been verified to be false) related to COVID-19 has spread quickly and widely via social media and has covered topics such as the latest developments of the crisis, the origin and severity of the pandemic, vaccines and treatments, transmission and prevention, government policies and disposal, travel restrictions, and conspiracy theories [3,4]. The impact of COVID-19 misinformation can be destructive [5], causing irrational behavior, anxiety and fear, self-prescription of medication, media skepticism, and political apathy [6,7,8]. It has also reduced citizens’ acceptance of official statements, decreased trust in government services and institutional systems, and threatened the entire social system [9]. Ultimately, the spread of misinformation can cause more damage than the crisis itself, creating extra pressure for government agencies to dispute inaccuracies [10].

The combating of COVID-19 misinformation has become a serious challenge facing all countries and international communities [4]. Fact-checking is one important measure that governments can use to deal with the spread of misinformation, publicly reporting the accuracy of claims or texts that have been circulated through traditional media and social media [11]. During the pandemic, government agencies, independent media outlets, civic organizations, social groups, social media platforms, and technology giants, have all actively participated in fact-checking activities [12,13]. Social media organizations, such as Tencent and Facebook, have integrated fact-checking mechanisms into their platforms, while international news outlets, such as the BBC (United Kingdom), Alt News (India), and AFP Factuel (France), have launched evidenced-based services that examine the facts and claims behind stories shared on social media. In China, government agencies have dynamically released COVID-19 fact-checks through their official social media accounts [14].

The positive effects of fact-checking to combat COVID-19 misinformation have been verified by some studies [15,16,17]. Citizens who are exposed to fact-checks are found to have more negative comments about the misinformation and believe that others are more susceptible to it, thereby reducing their sharing intention [16]. Online experimental evidence demonstrated that fact-checking labels can effectively reduce the negative impact of misinformation related to the vaccine [17].

The key challenge to combating misinformation is how to provide citizens with greater access to fact-checking information [11]. Citizens that consume misinformation rarely notice the corresponding fact-checks simultaneously [18]. Studies have confirmed that fact-checks were shared less frequently than related misinformation during the COVID-19 pandemic [15]. Similarly, the speed in which fact-checks are shared is slower than that of misinformation [19]. The effectiveness of fact-checking is therefore greatly weakened, meaning that “better fact-checking campaigns may be required to increase the virality of fact-checking content for increasing its shareability” [15].

Existing studies have mainly focused on the effectiveness and efficiency of fact-checking as an activity [11,20], but lack attention on how to promote the sharing of fact-checks [11]. Some scholars have investigated the factors that affect the sharing of political fact-checks, but have mainly emphasized the role of party characteristics, individual need for orientation, and ideological intensity [11,21]. For example, based on both Facebook data and survey data, one study found that those people who have moderate need for orientation, often seek political information through social media, and show liberal tendencies, are more likely to share fact-checks when discussing politics on Facebook [11]. Twitter data from the 2012 presidential election in the United States also demonstrated that parties tend to share fact-checks that favor their candidates and discredit competitors [21]. This study aims to systematically examine what triggers citizens to share COVID-19 fact-checks posted by the official social media accounts of Chinese governments on Sina Weibo. Considering that the elaboration likelihood model (ELM) has been gradually introduced by scholars to analyze social media communication behaviors in recent years [22,23], this study focuses on the role of various cues of fact-checking posts, including content-related central cues, such as content importance and theme, and peripheral cues, including media richness, source credibility, social media strategy, and social media capital.

## 2. Literature Review

### 2.1. The Elaboration Likelihood Model

The ELM is a dual process theory that focuses on the formation and changes in individuals’ attitudes [24]. The theory posits that when individuals are exposed to external information, the way in which they process the information directly affects their attitudes and behavioral changes, thereby influencing persuasive effects. It suggests that individuals adopt two approaches when processing information, namely, the central route and the peripheral route. With the central route, people exert themselves to process information. Through elaborate thinking, individuals adopt multiple standards to process content-related central clues, and judge and evaluate the quality of information to create a rational response and form a lasting and stable attitude. With the peripheral route, individuals tend to lack the motivation, or the ability to consider carefully, or spend too much cognitive effort in deciphering the information. Attitudes are formed by emotional transfer or easily obtainable peripheral cues, such as source credibility, and are relatively short-lived and vulnerable. The ELM theory has been widely employed in information dissemination and consumer behavior studies, and more recently has been used in research that explores social media dissemination behaviors [22].

An individual’s sharing behavior on social media is often the result of persuasion, which is closely related to how people process and evaluate online posts [23]. In the context of public health crises, audiences may employ both central and peripheral routes to process information contained in social media posts [25]. Firstly, social media has become an indispensable platform for individual expression. During crises, posts dynamically update and multiply quickly. The resulting overabundance of information makes audiences feel that they lack the time and ability to carefully review posts’ details, and instead focus on other peripheral cues, such as the number of fans and visual elements, such as attached pictures or videos [23]. Secondly, individuals may possess stronger information needs than in normal situations. These needs will prompt them to focus on content-related central cues to seek satisfaction and adopt the central route to process information [26]. Hence, both central and peripheral cues may influence the number of shares that COVID-19 fact-checks receive. This means that the ELM has potential advantages for studying the behavior of Chinese citizens’ when sharing fact-checks via social media. It provides a micro-perspective to examine how various related factors affect citizens’ sharing behavior by classifying them into two types, namely central and peripheral cues [25]. Moreover, we can investigate the relative importance of central and peripheral cues to further clarify the influence process itself [25].

### 2.2. Research Hypotheses and Model

**Figure 1:**
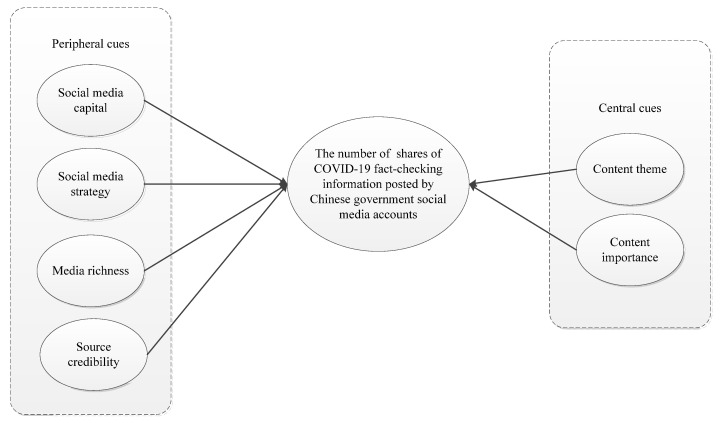
The theoretical model of the study.

According to the ELM, when individuals view COVID-19 fact-checks, released by official government social media accounts, they will refer to the corresponding central and peripheral cues to process the information, and then decide whether to share it or not. Based on the integration of existing research, this study advocates that peripheral cues include media richness [22,26], source credibility [27,28], social media capital [25], and social media strategy [29], while central cues include content theme and content importance [25,30,31]. To conclude, this study developed the theoretical framework shown in Figure 1.

#### 2.2.1. The Effect of Peripheral Cues on the Sharing of Fact-Checks

##### Social Media Capital

Social media capital refers to “the stock of social media-based social resources an organization has generated via its social media efforts” [31]. These resources include the size of the online network cultivated by the organization through social media activities, the strength of the relationship, and the centrality of the organization in the network, and so on. Social media capital can be obtained through various activities, such as sending messages and establishing connections [31]. The distribution of social media capital follows a power law, while different organizations have varying social media capital. Although researchers sometimes dispute how to measure social media capital, the number of followers is still regarded as one of the key indicators [31,32]. The 820 COVID-19 fact-checks analyzed in our study came from 413 Sina Weibo accounts of Chinese governments with different numbers of followers. Prior studies have demonstrated that the number of followers positively affects the number of reposts [33]. For instance, the number of followers of a Twitter account positively affected the number of reposts about breast cancer tweets [34], and its influence was even greater than other variables, such as the content itself and media richness. Therefore, the more followers that government social media accounts have, the stronger their social media capital and online social influence is, and the greater the possibility of fact-checks being reposted. Therefore, hypothesis 1 is as follows:

**Hypothesis** **1** **(H1).** *Social media capital positively affects the number of shares of COVID-19 fact-checks posted by Chinese Government social media accounts.*

##### Social Media Strategy

Government agencies use different strategies in operating their official social media accounts, based on varying organizational tasks, goals and arrangements [35,36,37]. In 2010, government social media strategies were summarized into push, pull, and networking strategies while, in 2012, scholars widely incorporated the transaction strategy. Three of them, push, pull and networking, are now widely adopted by researchers [38,39,40,41].

The push strategy regards citizens as audiences of governments and social media as a supplementary channel for broadcasting information [35,36]. The strategy is frequently employed due to its low cost and alignment with the mission of most government agencies: to disclose information and educate citizens [36,42]. The use of social media, based on the push strategy, involves almost no interaction with the public, while its purpose is to push information to citizens [35,37]. The pull strategy treats citizens as a sensor for governments and social media, and as a channel for input [35]. Pull strategies usually involve attracting citizens to government websites, inviting them to vote or to provide information and suggestions such as identifying suspects or reporting burglaries [38,39,41]. The networking strategy posits that citizens are co-producers of government policies and services and regards social media as an interaction channel [35,36]. Networking strategies include expressions of gratitude and appreciation, appealing to the public to follow the social media accounts of partner agencies, using super topics, and responding to private messages [38,39,41]. It can also promote the formation of long-term trust between governments and their citizens [35,39].

Previous studies have discussed the impact of social media strategies on citizens’ sharing behaviors towards Government social media accounts, however the results are controversial. Most have found that posts employing the pull strategy receive a greater number of shares, compared with those following the push strategy [29,38,41]. However, a recent study into 9873 tweets posted on 16 municipal police station Twitter accounts in the United States found that the difference between the number of retweets of push versus pull posts was not significant [39]. Therefore, Hypothesis 2 is as follows:

**Hypothesis** **2** **(H2).** *Compared with the push strategy, the pull strategy and networking strategy positively influence the number of shares of COVID-19 fact-checks posted by Chinese Government social media accounts.*

##### Media Richness

Media richness refers to the capacity of media to act as an information carrier, emphasizing its ability to promote meaning [43]. The development of new media technologies make it easier for people to create, disseminate and use multimedia content, which has been widely linked to developments in social media. Social media posts are usually presented in plain text or with accompanying pictures or videos. Plain text is viewed as the lowest in media richness, while pictures are moderate, and videos are the highest [44,45]. Due to its word limit restriction, Twitter users often add Supplementary Materials, such as images and videos, to extend the meaning of their posts [46]. Scholars have identified different results regarding the effect of media richness on the number of reposts or shares, with studies confirming that media richness can increase the number of retweets or shares of social media posts [47,48,49]. However, there are also inquiries that have concluded that pictures and videos have differentiating effects on the number of retweets [34,50]. Research into consumer companies Twitter accounts, from the automotive and luxury consumer goods sectors, confirmed that pictures improved the number of retweets, yet video posts had a negative effect [50]. By analyzing 1018 tweets relating to Breast Cancer Awareness Month, posts that included pictures were found to be positively associated with the number of retweets received, while the influence of video posts was insignificant [34].

This study posits that media richness will positively affect the number of shares of COVID-19 fact-checking information, posted by Chinese Government social media accounts. Compared with plain text, pictures and videos are more capable of stimulating different senses and attracting users’ attention, thereby increasing the trend of individuals to browse content [51]. Social media users can interpret posts with high media richness at a low cognitive cost [52], while content with high media richness can strengthen users’ trust [53]. Further, “our brains implicitly trust visual modalities such as images and video more than text because those modalities cue the realism heuristic (p. 157)” [54]. For computer-mediated communication, pictures and videos have richer visual sensory cues that increase presence and improve the efficiency of information presentation; in turn, this leads to a higher level of social media sharing [22]. The visual presentation represented by pictures and videos can also trigger emotions, attracting the attention of users to promote sharing behaviors [55]. Therefore, Hypothesis 3 is as follows:

**Hypothesis** **3** **(H3).** *Media richness positively influences the number of shares of COVID-19 fact-checks posted by Chinese Government social media accounts.*

##### Source Credibility

Source credibility indicates “the extent to which an information source is perceived to be believable, competent, and trustworthy by the information recipient” [56]. Citizens consider trustworthy information providers to be knowledgeable, honest, and genuinely interested in doing the right thing for their audience. Thus, more credible sources of information are more persuasive [57]. When public health crises occur, information spreads quickly and widely across social media platforms. In addition, platforms usually lack an effective emergency information recommendation system, which significantly increases the difficulty for individuals to obtain valuable messages from massive amounts of information [58]. When individuals believe that the source of information is reliable, their risk perception of the content is reduced [59]. When individuals deem that social media posts are from individuals or organizations with high credibility, they also tend to affirm the value of these posts [27]. To obtain valuable information with lower risk, individuals are more inclined to accept messages from high-confidence sources [58]. Reposting social media posts published by highly credible sources will also make citizens look more knowledgeable, considering that credibility usually means a high probability of correctness [60]. Extant studies have confirmed that the credibility of an information source positively affects an individual’s reposting behavior [60,61].

The COVID-19 fact-checking posts, which this study examines, were published by the official Sina Weibo accounts of 413 Chinese Government agencies. The agencies were divided into Central Government agencies and local government agencies. Scholars have indicated that, compared with local governments, Chinese citizens hold more trust towards central governments [62,63]. Hence, the following hypothesis is proposed:

**Hypothesis** **4** **(H4).** *Compared with local government agencies, COVID-19 fact-checks posted by the official social media accounts of central governments are easier to be shared.*

#### 2.2.2. The Effect of Central Cues on the Sharing of Fact-Checking Information

##### Content Theme

The different effects of content themes on social media information sharing have been confirmed in previous studies [26,30]. For videos related to COVID-19, posted by the TikTok account of the National Health Commission of China, content referring to guidance and government actions were more likely to be reposted, compared with posts that showed appreciation towards frontline emergency services [30]. For COVID-19 information released by the Sina Weibo account of the National Health Commission of Chin, latest developments of the pandemic and government actions were easier to promote citizen engagement (calculated by the sum of reposts, comments, and likes) [26]. The diverse COVID-19 information posted on social media has led to relevant fact-checks that cover multiple themes, including prevention and dissemination, the latest developments of the pandemic, and government decisions and actions [12]. Considering that individuals have various requirements for receiving COVID-19 information, the fact-checks of various themes may also meet the needs of the audience at specific moments to different degrees. The problem is that the degree of satisfaction of needs directly determines citizens’ subsequent attitudes and behaviors. Research has confirmed that the likelihood of posts being reposted varies greatly due to the different ability and degree of content themes to satisfy individuals’ needs [25,30]. Therefore, Hypothesis 5 is as follows:

**Hypothesis** **5** **(H5).** *Content theme has significant differential effects on the number of shares of COVID-19 fact-checks posted by Chinese Government social media accounts.*

##### Content Importance

Content importance emphasizes the degree of intrinsic importance that one fact-check possesses [24], dependent on situational relevance. Important fact-checks are more likely to satisfy an individual’s information needs and reduce uncertainties in comparison with ordinary ones. Although existing studies lack direct investigation into the relationship between the importance of fact-information and citizens’ reposting behaviors, scholars have paid attention to the effect of content importance on social media information reposting behavior. By analyzing 1872 tweets related to #BlackLivesMatter, content importance was found to be positively affected the number of retweets received. Their study also found that the likelihood of reposting increases when important tweets contain emotional narratives [64]. An online experimental study into 660 Korean adults showed that perceived message importance promoted individuals’ willingness to verify and share rumors about influenza vaccines [65]. Content importance was also proved to be a critical factor that drove Twitter users to share disaster information during crises. During disasters, individuals are also more likely to repost when they recognize the importance of messages [66]. Thus, this study posits that content importance can promote the sharing of COVID-19 fact-checks. Therefore, Hypothesis 6 is as follows:

**Hypothesis** **6** **(H6).** *Content importance positively promotes the number of shares of COVID-19 fact-checks posted by Chinese Government social media accounts.*

## 3. Materials and Methods

### 3.1. Data Collection

This study examines why citizens share COVID-19 fact-checking information, posted by the official social media accounts of Chinese governments, using data publicly available on Sina Weibo. In response to the widespread surge of misinformation posted to Sina Weibo, the platform created an official account called “Weibo Refutes Rumors” on 18 November 2010, to combat the spread of misinformation. The account actively promotes fact-checking activities by reposting relevant content published by various official accounts on Sina Weibo, including accounts managed by companies, governments, and media agencies. It also acts as an important channel for reporting misinformation on the Sina Weibo platform. In addition, the account occasionally releases original posts to refute misinformation. In China, during the COVID-19 outbreak, it played an important role in facilitating the dissemination of fact-checking information. It not only reposted fact-checks from various official accounts but, regularly pushed fact-checked information to its users.

This study, therefore, collected data from the “Weibo Refutes Rumors” account. First, according to the timeline of the COVID-19 outbreak, it was identified that the first COVID-19 fact-checking post was released by the official Sina Weibo account of the Wuhan Municipal Government Agency on 1 January 2020, titled “Wuhan Release”. Then, based on the “Weibo Refutes Rumors” account, all related posts, published by the official accounts of Chinese governments, were manually collected from 1 January to 6 April 2020. In total, 820 valid fact-checking posts were obtained. These posts came from 413 Chinese Government agencies’ official accounts. The dataset included government social media account names and the government agency to which it belonged. In addition, the number of followers, number of reposts, the multimedia attributes of each post (i.e., text, pictures or videos), the complete text of each post, and the “importance” tags automatically labelled by Sina Weibo, were collected for analysis.

### 3.2. Operationalization of Variables

The number of shares of COVID-19 fact-checks posted by Chinese Government social media accounts and the number of reposts of the 820 fact-checks were collected through Sina Weibo and used as objective data to measure the dependent variable.

Social media capital. By referring to previous study [32], the number of followers of government Sina Weibo accounts was used to measure social media capital.

Social media strategy. This study divided social media strategies into push, pull, and networking [38,39]. When posts involved “punishment announcement”, “policy statements” or “situation announcement”, it was classified as a push strategy. If the post purported to “help reporting rumors”, “posting a poll or survey” or “links to the department’s website or other sites”, it was classified as pull strategy. In instances when indicators in posts included “support and appreciation”, “using the function of super topic”, “responding to questions” or “requesting to follow Weibo accounts and retweet”, they were classified as networking strategy. Push, pull, and networking strategy were coded as 1, 2 and 3, respectively.

Media richness. Following previous study [26], media richness was divided into three categories: low, moderate, and high. Low media richness refers to plain text without any attached multimedia content. Moderate media richness refers to posts containing pictures or GIFs, while high media richness included posts that contained videos. The coders respectively used 1, 2 and 3 to represent low, moderate, and high media richness.

Source credibility. The 820 fact-posts were obtained from the official Sina Weibo accounts of 413 Chinese governments. Some accounts belonged to central government agencies, while some were managed by local government agencies. Research suggested that, compared with local governments, people show more trust towards the Central Government in China [62,63]. Therefore, when measuring source credibility, the fact-checks posted by the Sina Weibo accounts of Central Government agencies were marked as “1”, while those posted by local government agencies were marked as “0”.

Content theme. With reference to previous research [12], this study divided fact-checking posts into four categories: latest COVID-19 news, government actions towards COVID-19, pathology and treatment of COVID-19, and social conditions during COVID-19. These four categories were marked as “1”, “2”, “3” and “4”, respectively. The codebook in Table 1 contains examples.

Content importance. This study collected data from the “Weibo Refutes Rumors” account which was created to combat misinformation sharing on the Sina Weibo platform. During the COVID-19 crisis, it used a tracing mechanism to identify and repost fact-checks related to the pandemic, released by various accounts on Sina Weibo, such as traditional media agencies, companies, and government agencies. Further, it used the internal algorithm mechanism to label some posts with “important” tags. In this study, posts labelled as an “important message” (i.e., important fact-checking content) were coded as “1”, while others were coded as “0”.

### 3.3. Inter-Coder Reliability and Data Analysis

Two postgraduate students were employed to complete the coding work. It took 2 h to train the coders to learn the coding norms. To ensure inter-reliability, the coders coded 20% of the sample randomly and independently as a pretest. The results were as follows: The Kappa value with “media richness”, “source credibility”, and “content importance” was 1; the Kappa value for social media strategies was 0.881. The Kappa value for content theme was 0.892. These results show that the inter-reliability was high enough to be accepted. Given that the number of shares is count data, and the distribution of shares (Min = 1, Max = 32,976, M = 149.29, SD = 1326.32, Skewness = 20.16, Kurtosis = 471.21) is over-dispersed, negative binominal regression is more appropriate for conducting analysis. By using a negative binominal regression model, the impact of peripheral cues and central cues on the number of shares was estimated. All analyses were conducted using STATA version 15.0.

## 4. Results

### 4.1. Descriptive Analysis

Among the 820 fact-checks obtained, the number of shares of the fact-checking posts showed a huge variation, with 33.05% of them being shared less than 10 times, while 16 fact-checks (5.93%) were shared over 1000 times. The most prevalent theme of fact-checks related to the latest COVID-19 news (*n* = 375, 45.7%), followed by Government action towards COVID-19 (*n* = 365, 44.5%), social conditions during COVID-19 (*n* = 44, 5.4%), as well as pathology and treatment of COVID-19 (*n* = 36, 4.4%). Only 26 (3.2%) fact-checks were from Central Government social media accounts, while all others (*n* = 794, 96.83%) came from local government social media accounts. On average, central government social media accounts have a higher number of followers (M = 9,765,156.54, SD = 5,459,693.56) than local government agency accounts (M = 530,397.36, SD = 1,359,064.88). More than 80 percent of fact-checks (*n* = 673) attached photos to convey information. A total of 124 were presented as plain text only, while videos were attached less frequently (*n* = 23, 2.8%). As for social media strategy, most of the fact-checking posts used the push strategy to convey information (*n* = 685, 83.1%), followed by the pull (*n* = 110, 13.3%) and networking strategies (*n* = 25, 3.0%). In total, 59 fact-checks (7.2%) were labelled as ‘important’.

### 4.2. Hypotheses Test

Table 2 shows the results of the negative binominal regression model which predicted the number of shares of the COVID-19 fact-checks. From the perspective of peripheral cues, H1 posited that fact-checks posted by government social media with high levels of social capital were more likely to be shared by social media users. As shown in Table 2 (Model 1), social media capital is positively associated with the number of shares of COVID-19 fact-checking posts. The incident rate ratio (IRR) value shows that a one-unit increase in the number of followers would lead to an increase in the number of shares by a factor of 0.26 (IRR = 1.26, *p* < 0.001). Therefore, H1 was supported.

H2 proposed that, compared with the push strategy, pull strategy and networking strategy would positively influence the number of shares of fact-checking posts. Given that the social media strategy is a categorical variable, the push strategy was treated as the reference group. Results (Model 1) show that the pull (IRR = 1.35, *p* = 0.036) and networking strategy (IRR = 2.65, *p* < 0.001) both positively related to the number of posts shared. This means that, in comparison to the push strategy, fact-checks that use of the networking strategy leads to a 165% increase in the number of shares, while those employing the pull strategy obtained a 35% increase in the number of shares. Thus, H2 was supported.

H3 suggested that media richness positively influences the willingness of citizens to share fact-checking posts. As Model 1 shows, a one-unit increase in the level of media richness would produce an average of 0.65 shares. (IRR = 1.65, *p* < 0.001). Thus, H3 was supported.

H4 stated that fact-checks posted by government social media accounts of central government agencies are more likely to be shared than those posted by local governments. Our results (Model 1) show that source credibility was positively associated with the number of shares (IRR = 2.24, *p* = 0.002). This means that, compared with fact-checks posted by local governments, fact-checks posted by central governments lead to an increase in the number of shares by a factor of 2.24. Thus, H4 was also supported.

From the perspective of central cues, H5 argued that the number of shares is contingent upon the content theme. Latest COVID-19 news was treated as the reference group since content theme is a categorical variable. Results show that fact-checks related to government action was negatively related to the number of shares (IRR = 0.75, *p* < 0.001), whereas fact-checks related to social conditions were positively associated with the number of shares (IRR = 2.18, *p* < 0.001). This means that, in comparison to the latest news, fact-checks about government action leads to a 25% decrease in the number of shares, but fact-checks related to social conditions, results in a 118% increase. However, the relationship between fact-checks related to medical issues associated to COVID-19 and the number of shares is not significant. Thus, H5 was supported.

H6 proposed that content importance positively predicts the number of shares. Results show that the importance of fact-checks is positively associated with the number of shares (IRR = 32.94, *p* < 0.001). Compared with those fact-checks not labelled with ‘importance’, ‘important’ fact-checks attracted more shares by a factor of 32.94. Thus, H6 was supported.

To compare the roles of peripheral cues and central cues in predicting the number of shares of the fact-checking posts, we entered these two clusters of factors separately into the model. Results are presented in Model 2 and Model 3. For negative binomial regression, though McFadden’s pseudo-R-squared (pseudo R^2^) cannot be interpreted as R^2^ in a linear regression, it is best used to assess the model goodness of fit [67,68]. Since higher values indicate better goodness of model fit, central cues (pseudo R^2^ = 7.21) play a more important role than peripheral cues (pseudo R^2^ = 3.30) in predicting the number of shares that fact-checks receive.

## 5. Discussion

### 5.1. Summary of Findings

By investigating the effects of peripheral cues (i.e., social media capital, social media strategy, media richness, and source credibility) and central cues (i.e., content theme and content importance) on the number of shares of COVID-19 fact-checks posted by Chinese Government social media accounts, this study provides some interesting findings.

First, our results suggest that social media capital positively influences the number of shares of COVID-19 fact-checks posted by official government social media accounts. This extends the conclusions of previous research to the context of public health crises. One study found that the number of followers as social capital could increase the number of reposts and comments under normal conditions [69]. Another study identified that more followers led to a higher level of online bonding social capital which created “a feeling of emotional support by like-minded people that are interested in one’s life and one’s opinions” [70]. Similarly, some studies found that posts published by social media accounts with a large number of followers were more likely to reach a wider audience, with the likelihood of being seen and reposted increasing [25]. The more followers, the stronger the “chain effect” of reposting: not only can followers of government social media accounts repost COVID-19 fact-checks, but the followers of these sharers may also repost them, spreading them more widely to their respective networks, thereby amplifying potential audiences [71].

Second, the results evidenced that media richness positively affects the number of shares of COVID-19 fact-checks posted by Government social media accounts. This result further confirmed the result of previous study which concluded that tweets that contain pictures or videos in crisis situations achieve a greater number of retweets [23]. Compared with plain text, pictures provide more visual cues, while videos stimulate citizens’ vision and auditory senses [22]. For social media meditated communication, posts with high media richness are capable of offering citizens a sense of presence in real-time, thereby promoting telepresence [22]. Posts with high media richness strengthen the efficiency of information presentation by producing stronger telepresence and arousing citizens’ emotions, thus achieving a higher level of engagement [22,55]. This finding is the opposite of Chen et al. [26] who investigated Chinese citizens’ engagement via government social media during COVID-19, identifying that media richness reduces engagement. Nevertheless, unlike the fact-checks issued by government social media accounts of Chinese governmental agencies at all levels, their study concerned all COVID-19-related posts on the official Sina Weibo account of the National Health Commission of China, including routine posts and fact-checking posts. This means that when exploring the effects of media richness during public health crises, the role of content themes, such as routine posts or fact-checking posts, should be fully considered.

Third, this study confirmed that both the pull and networking strategies promote the number of shares of COVID-19 fact-checks posted by government social media accounts, in comparison with the push strategy. Echoing with previous study, this finding extends the conclusions in the context of public health crises. One prior study studied 40 social media accounts of U.S. police agencies and confirmed that the pull strategy will receive more shares than the push strategy, under normal circumstances [38]. Another study analyzed 14 Facebook accounts of U.S. police agencies and found that pull posts obtain more shares compared with push ones [29]. This means that, regardless of whether it is a crisis or regular circumstances, government social media posts that adopt the pull strategy will receive more reposts; “the pull practice has shown its effectiveness in terms of being spread out quickly (p. 187)” [38]. The networking strategy employs social media as a tool to directly connect with citizens, encouraging the public to proactively participate in the information sharing process, such as through policy crowdsourcing [72]. The application of networking strategy can effectively stimulate communication between governments and citizens and encourage the public to participate in government policy creation and subsequent actions [72]. Networking strategies, such as support and appreciation, trigger emotional experiences to obtain interaction [39]. Moreover, the networking strategy is conducive to cultivating long-term trust between citizens and governments [35,39]. Governments that adopt the networking strategy when managing social media accounts pay greater attention to dialogue and emphasize extensive public discussions [40].

Fourth, the study showed that source credibility is a key driver for increasing the number of shares of COVID-19 fact-checks posted by government social media accounts. Specifically, fact-checking messages, posted by the official Sina Weibo accounts of central government agencies, are easier to be reposted than those posted by local government agencies. In China, citizens generally believe more in central governments rather than local governments during emergencies. This may be due to the “high diffuse support (for the party) at the central level and high specific support for a central government that is visible and often plays a leading role in crisis management, frequently supported by strong symbols (p. 389)” [63]. Consequently, citizens tend to trust the information posted by central government agencies on social media during public health crises. The efforts required and potential risks to repost these posts are the lowest, considering the explosion of COVID-19 information with uneven quality across platforms. The act of reposting credible posts not only benefits more people but also improve personal reputation.

Fifth, this study also demonstrated the differentiated effects of content themes on the number of shares of COVID-19 fact-checks posted by government social media accounts. Compared with fact-checking posts related to the latest COVID-19 news, posts concerning social conditions during COVID-19 are more likely to be reposted; while posts providing insights to government actions significantly reduce the number of reposts. Posts related to pathology and treatment negatively affects reposts, although the result is insignificant. Social condition posts mainly involve fact-checks about the shortage of living materials. The impact of COVID-19 on citizens’ lives seems to be more direct and specific and is easier to be noticed and reposted. This research generally supports the prior findings that the effects of content types on the number of reposts are different. The main reason for this is that citizens have diversified needs for COVID-19 related information. When a post meets the needs of an individual, or an individual speculates that it may meet the needs of others, he or she is more likely to repost [26,30].

Sixth, our study found that content importance can increase the number of shares of COVID-19 fact-checks posted by government social media accounts. This research extends the results of prior research to the context of public health crises, that is, important tweets are more likely to be reposted [64]. Content importance has been reflected in the theory of rumor transmission. Previous study emphasized content importance and content ambiguity as the core driving forces for rumor dissemination [73]. This study’s findings further confirm that content importance is critical to the dissemination of fact-checks. The more important a message, the deeper an individual’s involvement and the higher their motivation and possibility of reposting [74]. Since most citizens lack sufficient resources and motivation to pay careful attention to all fact-checks, they usually gather and process information selectively; “individuals do not have to be cognitively highly sophisticated to form attitudes regarding issues they consider personally important” [75]. Thus, citizens tend to focus more on important information, thereby increasing the possibility of reposting.

Lastly, this study confirmed that central cues are more effective in promoting the sharing of fact-checks than peripheral cues. During the public crisis, the social context became highly uncertain, and stakeholders, such as citizens, have held an unprecedented desire for accurate and useful information [26]. When COVID-19 fact-checks can satisfy the needs of citizens and eliminate panic and anxiety caused by misinformation, in a timely manner, they will show more active engagement in government social media, including information sharing and other interactive behaviors. Therefore, government agencies should fully evaluate the content theme, content importance and other factors related to the central cues, and accurately push the fact-checks most needed by the public, when releasing fact-checks through their official social media accounts. This will promote citizens spontaneous sharing behavior, thereby allowing more people to have access to this information and enhance the effectiveness of fact-checking. After clarifying central cues, government agencies can then consider which social media and what strategies to use to push these fact-checks, to further amplify the dissemination effect.

### 5.2. Practical Implications

This study offers several valuable insights for misinformation correction by government social media accounts and platform providers during public health crises. First, government social media accounts are essential tools in combating the spread of misinformation during crises. The networking feature of social media allows citizens access to fact-checking information. Government agencies, especially central government agencies, are expected to open official accounts across social media platforms and proactively publish fact-checking posts. Citizens typically trust fact-checks posted by the official accounts of central governments more, and actively engage in reposting them. Additionally, greater attention should be paid to the accumulation of followers when operating government social media accounts. The number of followers as important social media capital significantly promotes the dissemination of social media posts.

Second, government social media accounts should continually use both pull and networking strategies to release fact-checking information. Compared with the one-way push strategy, both the pull and networking strategies can promote the sharing of fact-checking posts. For example, expressions of appreciation and support can enable citizens to perceive the value of actions which triggers reposting behaviors. The networking strategy requires government agencies to respond to public questions and interactions in a timely manner, promoting a dialogue loop and maximizing the communication effect of fact-checking. Meanwhile, information released via government social media should stress the balanced use of the three strategies to achieve the best effects [38].

Thirdly, government agencies should attach great importance to the role of multimedia content when publishing fact-checking information. Further, government social media accounts should employ a combination of pictures or videos with text to encourage its rapid diffusion and attract more public attention. Pictures or videos can not only provide more sensory cues but also arouse public emotions. These can increase citizens’ investment in posts, such as time, voluntary sharing, and other pro-social behaviors. Besides, multimedia content can also enhance citizens’ trust in fact-checking. Therefore, posts that contain multimedia content can easily increase citizen engagement in various activities initiated by government social media.

Lastly, government agencies should value the role of central cues, such as content themes and content importance. Specifically, when government agencies use official accounts to post fact-checks, they can attach importance to the demands of the public and their dynamic changes in different stages of the crisis; only in this way can appropriate fact-checks be released at the right time to meet the most urgent demands of citizens, and enhance the importance of this information and promote its dissemination across social media platforms.

### 5.3. Limitations and Future Directions

This study has several limitations. First, although it is feasible to adopt automatically labelled ‘importance’, through the algorithm of Sina Weibo as the measurement of the importance of fact-checks, future studies may enrich the evaluation indicators of content importance through qualitative techniques, such as questionnaires. Second, this study focused on the context of China only, while European and American countries were omitted and could be further investigated. Unlike East Asian countries, such as South Korea and China, which emphasize collectivism, Western countries, such as the United States and United Kingdom, advocate individualism. Cultural values and norms affect the design, perception, and use of social media [72]. Facebook and Twitter reflect the popular values of Western culture which are somewhat different from Sina Weibo. One study found that Korean government agencies presented collective cooperation when using Twitter, while US governments paid more attention to their own goals [72]. It is also necessary to investigate the specific mechanisms that promote the number of shares of fact-checking information posted by government social media accounts in Western countries during crises. Third, this research focused on the context of public health crises. Whether the influencing mechanism is applicable to other types of crises, such as natural disasters and man-made accidents, is still unknown and requires further investigation. Fourth, this study is based on Sina Weibo, similar in functionality to Twitter, but questions remain whether the results would be similar on Facebook and other social media platforms. Studies have shown that police departments’ social media strategies on the two platforms are different, such as Facebook prefers the push strategy while Twitter prefers the networking strategy [38].

## 6. Conclusions

In December 2019, the outbreak of a novel type of coronavirus, later renamed COVID-19 by the WHO, caused mass panic, fear and anxiety among the world’s citizens. In this time of uncertainty, people flocked to social media to seek health-related information related to the disease. Concurrently, large amounts of misinformation began to spread quickly and widely across social media platforms in China, such as Sina Weibo. To refute misinformation, Chinese government agencies published official information through traditional media channels. Meanwhile, they released fact-checking posts via their official accounts on Sina Weibo to dispute inaccurate information. However, the effect of this approach is dependent on the breadth of fact-check diffusion. Hence, citizen engagement in the sharing of COVID-19 fact-checking content, posted by official government social media accounts, is paramount to minimizing the sharing of misinformation. This study took Chinese governmental agencies’ actions in refuting COVID-19 misinformation, through their government social media accounts, as an example. Based upon ELM model, this study finds that both peripheral cues and central cues play important roles in the sharing of fact-checks. Findings in this study can offer valuable insights for misinformation correction by government social media accounts and platform providers during public health crises.

## Figures and Tables

**Table 1:** Codebook examples of content theme.

Categories	Example Posts
Latest COVID-19 News	#Win the fight against the epidemic# Newly confirmed cases in Siping spread online as a rumor!!
Government Action towards COVID-19	【Fact-checks】Fake! The notice of the Shandong Provincial Department of Education on matters related to the start of the 2020 spring school is a rumor.
Pathology and Treatment of COVID-19	Can zinc treat the new coronary pneumonia? Rumor! Don’t believe it! #The police will not retreat before the epidemic# #Public security in action to fight COVID-19# #fact-checking on Sina Weibo#
Social Conditions during COVID-19	【Urgently refute rumors! Ezhou citizens must not follow the trend to buy grain and oil!】

**Table 2:** Predicting the number of shares of COVID-19 fact-checks.

	Model 1		Model 2		Model 3	
	IRR	SE	IRR	SE	IRR	SE
(Intercept)	1.01	0.34	2.10 *	0.78	57.14 ***	4.03
Peripheral cues						
Social media capital	1.26 ***	0.02	1.39 ***	0.03		
Social media strategy (Reference group: Push)						
Pull	1.35 *	0.19	5.19 ***	0.91		
Networking	2.65 ***	0.66	2.14 *	0.69		
Source credibility	2.24 **	0.59	0.84	0.28		
Media richness	1.65 ***	0.19	0.90	0.12		
Central cues						
Content theme (Reference group: Latest COVID-19 News)						
Government Action towards COVID-19	0.75 **	0.07			0.55 ***	0.06
Pathology and Treatment of COVID-19	0.89	0.19			0.75	0.18
Social Conditions during COVID-19	2.18 ***	0.43			2.12 **	0.46
Content importance	32.94 ***	5.62			41.67 ***	7.88
Log likelihood	−3898.87		−4201.54		−4044.54	
Pseudo R^2^(%)	10.26		3.30		7.21	

Note. * *p* < 0.05; ** *p* < 0.01; *** *p* < 0.001.

## Data Availability

Please contact authors for data.
